# Selection of the *N*-Acylhomoserine Lactone-Degrading Bacterium *Alteromonas stellipolaris* PQQ-42 and of Its Potential for Biocontrol in Aquaculture

**DOI:** 10.3389/fmicb.2016.00646

**Published:** 2016-05-09

**Authors:** Marta Torres, Esther Rubio-Portillo, Josefa Antón, Alfonso A. Ramos-Esplá, Emilia Quesada, Inmaculada Llamas

**Affiliations:** ^1^Faculty of Pharmacy, Department of Microbiology, University of GranadaGranada, Spain; ^2^Biomedical Research Centre (CIBM), Institute of Biotechnology, University of GranadaGranada, Spain; ^3^Department of Marine Science and Applied Biology, University of AlicanteAlicante, Spain; ^4^Department of Physiology, Genetics and Microbiology, University of AlicanteAlicante, Spain

**Keywords:** quorum quenching, aquaculture, *Vibrio*, biocontrol, *N*-acylhomoserine lactone

## Abstract

The production of virulence factors by many pathogenic microorganisms depends on the intercellular communication system called quorum sensing, which involves the production and release of signal molecules known as autoinducers. Based on this, new-therapeutic strategies have emerged for the treatment of a variety of infections, such as the enzymatic degradation of signaling molecules, known as quorum quenching (QQ). In this study, we present the screening of QQ activity amongst 450 strains isolated from a bivalve hatchery in Granada (Spain), and the selection of the strain PQQ-42, which degrades a wide range of *N*-acylhomoserine lactones (AHLs). The selected strain, identified as *Alteromonas stellipolaris*, degraded the accumulation of AHLs and reduced the production of protease and chitinase and swimming motility of a *Vibrio* species in co-cultivation experiments *in vitro*. In the bio-control experiment, strain PQQ-42 significantly reduced the pathogenicity of *Vibrio mediterranei* VibC-Oc-097 upon the coral *Oculina patagonica* showing a lower degree of tissue damage (29.25 ± 14.63%) in its presence, compared to when the coral was infected with *V. mediterranei* VibC-Oc-097 alone (77.53 ± 13.22%). Our results suggest that this AHL-degrading bacterium may have biotechnological applications in aquaculture.

## Introduction

Over the past decades, aquaculture has grown at an impressive rate, mainly due to the global decline of world fish supplies and to fill human demand (Food and Agriculture Organization of the United Nations [FAO], 2014). Bacterial diseases are among the most critical problems in commercial aquaculture and are caused in many cases by the proliferation of opportunistic bacteria, such as *Vibrio* spp.. This proliferation if due to the high animal densities and increases organic matter content of these systems (Defoirdt et al., 2007; Ruwandeepika et al., 2012). Classic antibiotics (e.g., those affecting essential bacterial processes, such as cell-wall synthesis, DNA replication, and protein synthesis) have been used in many countries to control bacterial outbreaks. Nevertheless, this widespread use of antibiotics eventually has led to antibiotic resistance by fish pathogens (Brown and Tettelbach, 1988; Akinbowale et al., 2006; Agersø et al., 2007; WHO, 2014). This serious situation has promoted the exploration of novel strategies for controlling marine pathogenic bacteria in aquaculture systems, such as immuno-stimulants, vaccines, water disinfection and probiotics, which are currently under analysis (Defoirdt et al., 2011). More recently one promising alternative is the inhibition of the expression of virulence genes that are regulated in many aquaculture pathogens by bacterial cell-to-cell signaling process known as quorum sensing (QS; González and Keshavan, 2006; Dong et al., 2007; Bjarnsholt et al., 2010; Natrah et al., 2011). However, it has recently been demonstrated that bacteria can also develop resistance mechanisms to compounds that interfere with QS (Defoirdt et al., 2010; Maeda et al., 2012; García-Contreras et al., 2013, 2016; Kalia et al., 2014).

Quorum sensing is a population-density-dependent gene-expression mechanism, which involves the production, release and recognition of signal molecules known as autoinducers. QS is an ubiquitous phenomenon in bacteria (González and Marketon, 2003; Ng and Bassler, 2009; Parker and Sperandio, 2009; Tait and Havenhand, 2013). Autoinducers include, amongst others, *N*-acylhomoserine lactones (AHLs) produced by the *Proteobacteria*; oligopeptides produced by the *Firmicutes*; and furanosylborate diester (AI-2) produced by both *Proteobacteria* and *Firmicutes* and used for interspecies communication (Whitehead et al., 2001; González and Marketon, 2003). QS regulates various phenotypes, such as biofilm formation, exopolysaccharide production, bioluminescence, conjugal DNA transfer, control of plasmid-copy number, virulence factors, and swarming, all of which have been shown to contribute to bacterial pathogenesis and have a significant impact upon human health, aquaculture, and the environment (Whitehead et al., 2001; González and Marketon, 2003; Parsek and Greenberg, 2005).

The disruption of QS can be performed by different mechanisms, such as the presence of some compounds that interfere with the detection of signal molecules (quorum-sensing inhibitors or QSIs); or signal degrading enzymes (Natrah et al., 2011). The first QSI compounds were halogenated furanones synthesized by the red marine alga *Delisea pulchra* (Givskov et al., 1996). These compounds have been reported to protect rainbow trout (Rasch et al., 2004), *Artemia* spp. (Defoirdt et al., 2006), and rotifers (Tinh et al., 2007) from vibriosis. Nevertheless, furanones cannot be used in practice due to the fact that the effective doses have proved to be toxic to many cultured organisms. More recently, several studies have demonstrated that natural products extracted from marine organisms are capable of inhibiting bacterial QS without causing toxicity and can be used for controlling biofouling communities (Skindersoe et al., 2008; Dobretsov et al., 2010, 2011; Linthorne et al., 2015). With respect to the second strategy, known as quorum quenching (QQ), many microorganisms belonging to the phyla *Actinobacteria, Bacteroidetes, Firmicutes*, and *Proteobacteria* produce enzymes that degrade AHLs, which are the main QS autoinducers in Gram negative bacteria (Uroz et al., 2009; Romero et al., 2010, 2011; Defoirdt et al., 2011; Torres et al., 2013). The QQ enzymes are classified into three major types according to their mechanisms: AHL lactonase (lactone hydrolysis), AHL acylase (amid hydrolysis) and AHL oxidase and reductase (oxidoreduction). Several studies have demonstrated the potential application of this latter strategy to control bacterial disease in aquaculture (Tinh et al., 2008; Nhan et al., 2010; Chu et al., 2014; Romero et al., 2014). In a previous study, we performed a search for AHL-degrading bacteria from a bivalve hatchery situated in Galicia, northern Spain, and characterized strain *Thalassomonas* PP2-459 since it had a high QQ activity (Torres et al., 2013).

In this study we have explored a fish hatchery located on the southern coast of Spain and studied the AHL-degrading capacity of 450 bacterial isolates. The QQ activity of these strains was tested with different synthetic AHLs. On the basis of our results, we selected a significant number of AHL-degrading bacteria and can report that the strain PQQ-42 reduced the bleaching process caused by *Vibrio mediterranei* VibC-Oc-097 in the coral *Oculina patagonica*.

## Materials and Methods

### Sampling Site and Processing

Samples were taken from six different tanks in a fish hatchery in Salobreña (Granada, Spain, 36°44′44.2′′N, 3°36′04.8′′W) in April 2013 and consisted of 100 mL seawater aliquots collected in polycarbonate tubes and taken immediately to the laboratory, where they were stored at 4°C until studied (always within 24 h). They were thoroughly homogenized by agitation and then serially diluted. Then, 100 μL of dilutions from 10^-4^ to 10^-7^ were surface-plated on marine agar (MA, Difco) and incubated at 25°C for 5 days. The medium and the incubation conditions used in this work were the same as those applied previously in studies carried out by our research group (Torres et al., 2013). A collection of 450 colonies, chosen on the basis of their different appearances, were re-isolated by streaking on a fresh medium and incubated in the same conditions described above.

### Bacterial Strains, Media, Compounds, and Culture Conditions

The aquaculture-related pathogenic *Vibrio* spp. used were the following: *V. anguillarum* ATCC 19264^T^, *V. nigripulchritudo* CIP 103195^T^, and *V. metschnikovii* NCTC 8483^T^, obtained from type culture collections, and *V. mediterranei* VibC-Oc-097 which was isolated from the coral *O. patagonica* (Rubio-Portillo et al., 2014). All the strains used in this study were cultured at 25°C in marine broth (MB, Difco) or in sterile filtered seawater (SFSW) supplemented with 0.1% (w/v) yeast extract (SFSWYE). *Agrobacterium tumefaciens* NTL4 (pZLR4; Chilton et al., 1974; Shaw et al., 1997) was cultured at 30°C in Luria Bertani (LB) medium (10 g tryptone, 5 g yeast extract and 10 g NaCl per liter) supplemented with 2.5 mmol L^-1^ CaCl_2_ × 2H_2_O and 2.5 mmol L^-1^ MgSO_4_ × 7H_2_O (LB/MC), in AB medium (3 g K_2_HPO_4_, 1 g Na_2_H_2_PO_4_, 1 g NH_4_Cl, 0.3 g MgSO_4_ × 7H_2_0, 0.15 g KCl, 0.01 g CaCl_2_, 0.0025 g FeSO_4_ × 7H_2_O and 5 g glucose per liter) or in MGM medium (11 g Na_2_HPO_4_, 3 g KH_2_PO_4_, 0.5 g NaCl, 1 g glutamate, 10 g mannitol, 1 mg biotin, 27.8 mg CaCl_2_ × 2H_2_O and 246 mg MgSO_4_ × 7H_2_O per liter) containing 50 μg gentamycin mL^-1^. *Chromobacterium violaceum* CV026 (McClean et al., 1997) and *C. violaceum* VIR07 (Morohoshi et al., 2007) were grown at 30°C in LB medium supplemented with kanamycin (50 μg mL^-1^).

The synthetic AHLs tested were the following: C_4_-HSL (*N*-butyryl-_DL_-homoserine lactone), C_6_-HSL (*N*-hexanoyl-_DL_-homoserine lactone), C_8_-HSL (*N*-octanoyl-_DL_-homoserine lactone), C_10_-HSL (*N*-decanoyl-_DL_-homoserine lactone), C_12_-HSL (*N*-dodecanoyl-_DL_-homoserine lactone), 3-oxo-C_12_-HSL (*N*-3-oxododecanoyl-_DL_-homoserine lactone) and 3-OH-C_10_- HSL (*N*-3-hydroxydecanoyl-_DL_-homoserine lactone; Sigma^®^).

### Screening for AHL-Degradation Activity Using Synthetic AHLs

The QQ activity of the 450 isolates was first tested in 96-well microtiter plates. Each of the bacterial strains was grown in 100 μL of MB (buffered to pH 7). After overnight incubation at 25°C, pH of cultures was adjusted to pH 7 again and each of the synthetic AHLs (C_6_-HSL and C_10_-HSL) were added in a final concentration of 10 μM and incubated at 25°C for 24 h. For the screening, 100 μL of LB agar 1.5% (w/v) was added to each well in microtiter plates and allowed to dry. Next, 20 μL of supernatant of cultures was spotted onto the agar surface and then a second layer of 50 μL of LB agar 1.5% (w/v) inoculated with each biosensor strain (5:1 v/v). The microplates were incubated for 24 h at 30°C (García-Aljaro et al., 2012).

A second assay was conducted with a well diffusion agar-plate as described elsewhere (Romero et al., 2011). Briefly, each of the synthetic AHLs (C_4_-HSL, C_6_-HSL, C_8_-HSL, C_10_-HSL, C_12_-HSL, 3-oxo-C_12_-HSL, and 3-OH-C_10_-HSL) were added in a final concentration of 10 μM to 500 μL of an overnight culture of each bacterial strain in MB medium (pH previously adjusted to 7) and incubated for 24 h at 25°C and at 100 rpm rotary shaking. The same quantities of AHLs were added to 500 μL of cell-free MB media and incubated in the same conditions as described above, as the negative controls. The remaining AHLs presented in each overnight culture were detected as follows. AB agar plates supplemented with 80 μg of 5-bromo-4-chloro-3-indolyl-β - D-galactopyranoside (X-Gal) and LB agar plates were covered with 5 mL of 0.7% (w/v) LB agar containing 500 μL of overnight cultures of biosensor strains. LB agar plates were used for *C. violaceum* CV026 and *C. violaceum* VIR07 overlays and AB agar plates with X-Gal were used for the *A. tumefaciens* NTL4 (pZLR4) overlay. Once the biosensor layer was dried, 6-mm-diameter wells were made in the surface of the medium with a sterile Pasteur pipette and 100 μL aliquots of culture supernatants were placed in each well. The plates were then incubated at 30°C for 24 h to check for the appearance of a colored halo around the wells. The strains that showed positive activity against all the AHLs tested were those that did not activate the biosensor strains and they were selected for further studies. We refer to them as AHL-degrading strains.

### Confirmation of AHL Degradation Activity by HPLC-MS

To confirm the QQ activity revealed in the well diffusion agar-plate assays by the AHL-degrading strains, we used high-performance liquid chromatography plus mass-spectrometry analysis (HPLC-MS; Romero et al., 2011). C_6_-HSL and C_10_-HSL were added to 500 μL samples of overnight cultures of AHL-degrading strains in MB medium to a final concentration of 10 μM and incubated for a further 24 h at 25°C and 150 rpm rotary shaking. The cultures were then centrifuged at 2,000 × *g* for 5 min, extracted twice with an equal volume of ethyl-acetate, evaporated under nitrogen flux at 50°C and suspended in 400 μL of acetonitrile for HPLC-MS analysis and quantification. As negative controls, AHLs (10 μM) were added to fresh MB medium and processed and extracted in the same way.

Analyses were carried out with a HPLC 1100 series (Agilent) equipped with a C8 pre-column (2.1 mm × 12.5 mm, 5 μm particle size) and a ZORBAX Eclipse XDB-C18 2.1 mm × 150 mm column (5 μm particle size) kept at 45°C. The mobile phase was composed of 0.1% v/v formic acid in water and 0.1% v/v formic acid in acetonitrile. MS experiments were conducted on an API 4000 triple-quadrupole mass spectrometer (Applied Biosystems, CA) equipped with a Turbolon source using positive-ion electrospray, multiple-reaction monitoring (MRM) mode. The MRM signals were used to generate relative quantification information by comparison with a calibration curve constructed for molecular-ion abundance, using each of the appropriate AHL synthetic standards.

### Location of the QQ Activity

In order to determine where was located the degrading activity in the selected strain PQQ-42, supernatant and crude cell extract (CCE) were obtained from a 24 h culture of the AHL-degrading strain. Fifteen milliliter of the overnight culture was centrifuged for 5 min at 2,000 × *g* to separate the cells from the culture media. The supernatant was filtered through a 0.22 μM pore-size membrane filter and kept at 4°C until use. The pellet was washed with 15 mL of phosphate buffered saline (PBS) pH 6.5, suspended in another 5 mL of the same buffer, sonicated intermittently in cold water bath (at a frequency of 35 kHz) for 5 min and centrifuged at 10,000 × *g* for 30 min at 4°C. The CCE obtained in this way was filtered through a 0.22 μM pore-size membrane filter and stored at 4°C (Romero et al., 2014). The protein concentration in the supernatant and CCE were determined by the method of Bradford (1976) with bovine serum albumin as the standard. The AHL-degradation activity in the cell-free culture medium and CCE was tested by incubating 500 μL of supernatant and CCE in the presence of each AHL (C_6_-HSL, C_8_-HSL, C_10_-HSL, C_12_-HSL, 3-oxo-C_12_-HSL, and 3-OH-C_10_-HSL) in a final concentration of 10 μM for 24 h at 25°C and 150 rpm, and the remaining AHLs were detected by well diffusion agar-plate as previously described.

### Identification of the QQ Enzyme

To determine whether the QQ activity was related, or not, to the hydrolysis of the lactone ring caused by lactonases, which enzymatically opens the lactone ring, we extracted AHLs from the reaction mixtures under acidic conditions following the procedure described in previous studies (Marketon et al., 2002; Llamas et al., 2005). For this purpose, AHLs (C_6_-HSL, C_8_-HSL, C_10_-HSL, C_12_-HSL, 3-oxo-C_12_-HSL, and 3-OH-C_10_-HSL) were added in a final concentration of 10 μM each to 500 μL of overnight cultures of the selected AHL-degrading strain PQQ-42 in MB medium and incubated for a further 24 h at 25°C and at 100 rpm rotary shaking. The mixtures were then centrifuged at 2,000 × *g* for 5 min, and the supernatants were acidified with HCl 1N to pH 2 and incubated for 24 h at 25°C and at 100 rpm (Yates et al., 2002; Uroz et al., 2005) rotary shaking before extracting the remaining AHLs as previously described. Finally, the remaining AHLs were suspended in LB medium and detected by well diffusion agar-plate assays using the corresponding biosensors.

### Study of QQ Activity Upon Crude AHL Extracts from Pathogenic Species of *Vibrio*

*N*-acylhomoserine lactones molecules were collected in crude extracts from 5 mL of cultures of pathogenic *Vibrio* species following the technique described in previous studies (Marketon et al., 2002; Llamas et al., 2005). Briefly, 10 μL of each crude extract was added to 5 mL of overnight cultures of the selected AHL-degrading strain PQQ-42 in MB medium, previously diluted 1:100, and incubated for 24 h at 25°C and at 100 rpm rotary shaking. The same quantity of crude extracts were added to 5 mL of cell-free MB medium and incubated for 24 h at 25°C as negative controls. The remaining AHLs were extracted twice with equal volumes of dichloromethane, dried, and finally suspended in 20 μL of 70% v/v methanol. To detect the AHLs, an overnight culture of the AHL biosensor strain *A. tumefaciens* NTL4 (pZLR4) was diluted 1:10 in 5 mL of the corresponding medium and poured onto AB medium supplemented with 80 μg X-Gal per mL. Once the overlay was dried, sterile paper disks 5 mm in diameter were placed onto them and 20 μL of each sample was applied. The plates were incubated overnight at 30°C to allow the biosensor organism to grow and surround the paper disks with blue haloes.

To confirm the QQ activity upon crude AHL extracts from the pathogenic species of *Vibrio*, the same samples were subjected to analytical and preparative thin-layer chromatography (TLC) as described in other studies (Marketon et al., 2002; Llamas et al., 2005). 20 μL of each sample and AHL standards were spotted onto a LCK-18 TLC plate (Whatman) and developed with 70% v/v methanol in water. The plate was air-dried and overlaid with top agar containing the *A. tumefaciens* NTL4 (pZLR4) biosensor strain before being incubated at 30°C. For the *A. tumefaciens* NTL4 (pZLR4) overlay, a 6–8 h culture in MGM medium was mixed with an equal volume of fresh medium, 1.5% w/v agar and 80 μg of X-Gal per mL.

### Co-culture Experiments

Co-cultivation experiments of the pathogenic species of *Vibrio* and the selected AHL-degrading strain PQQ42 were conducted in SFSWYE medium at different ratios. Briefly, an overnight culture of each *Vibrio* strain was added to an overnight culture of the strain PQQ-42 and incubated for 24 h at 25°C and at 100 rpm rotary shaking. We assayed six different ratios between each *Vibrio* sp. and the strain PQQ42. The ratios used were 10^2^ CFU mL^-1^ of *Vibrio* and 10^3^, 10^4^ or 10^5^ CFU mL^-1^ of PQQ-42, as well as 10^2^ CFU mL^-1^ of PQQ-42 and 10^3^, 10^4^ or 10^5^ CFU mL^-1^ of *Vibrio*. In each condition, the same concentration (CFU mL^-1^) of *Vibrio* was added to cell-free SFSWYE medium and incubated at 25°C as negative control. After 24 h of incubation, the remaining AHLs were detected and the effect on the production of virulence factors in *Vibrio* was analyzed. The concentration of *Vibrio* and PQQ-42 was also determined to evaluate whether they it was maintained during the co-culture assay. Ten-fold serial dilutions were prepared in SFSW and plated on Thiosulfate-Citrate-Bile-Sucrose (TCBS) agar and MA and incubated for 24 h at 25°C.

The remaining AHLs were extracted twice from each sample with equal volumes of dichloromethane, dried and finally suspended in 20 μL of 70% v/v methanol. To detect AHLs, an overnight culture of the biosensor strain *A. tumefaciens* NTL4 (pZLR4) was diluted 1:10 in 5 mL of the corresponding medium and poured onto AB medium supplemented with 80 μg X-Gal per mL. Once the overlay was dried, sterile paper disks 5 mm in diameter were placed on top of them and 20 μL of each sample was applied. The plates were incubated overnight at 30°C to allow the biosensor strain grow and surround the paper disks with blue haloes.

To evaluate the effect of the AHL degradation upon some of the virulence factors produced by *Vibrio* species, co-cultures were spotted on different media. Proteolytic activity was determined by spotting co-cultures in casein medium (Barrow and Feltham, 1993). Hemolytic activity was detected in blood agar medium (Barrow and Feltham, 1993). Chitinase activity was determined in marine broth supplemented with colloidal chitin (Wu et al., 2009). DNase activity was determined in DNase agar medium (Jeffries et al., 1957). Lipase activity was assayed in tributyrin agar and in marine agar supplemented with 1% (v/v) Tween 20 and Tween 80 (Sierra, 1957; Mourey and Kilbertus, 1976). Amylase activity was determined in marine agar supplemented with 1% (w/v) starch (Mourey and Kilbertus, 1976). Siderophore production was evaluated using chromoazurol S (CAS) reagents (Pérez-Miranda et al., 2007). Biofilm formation was measured using crystal violet assay and measuring absorbance at 540 nm (O’Toole and Kolter, 1998). The swimming motility test was carried out in soft agar (MB 0.3% w/v agar; Rui et al., 2008).

### Competition Assay with the Pathogenic Species of *Vibrio*

We evaluated the antagonistic activity of the selected AHL-degrading strain PQQ-42 upon the *Vibrio* species. An overlay of each *Vibrio* strain was prepared on MB agar plates. Then, a 6-mm-diameter well was made in each plate with the help of sterile Pasteur pipette. A 100-μL aliquot of the culture of PQQ-42 was poured into the well and incubated at 25°C for 24 h. The zones of inhibition around the well were recorded after the incubation. Wells containing only sterile MB were used as negative controls.

### Coral Infection Experiments

*In vivo* experiments were conducted with the coral *O. patagonica*, where *V. mediterranei* produces bleaching (whitening of tissue; Rubio-Portillo et al., 2014). Intact colonies of *O. patagonica* were collected in the Marine Protected Area of Tabarca on the Alicante Bay coast (Western Mediterranean Sea, Spain, 38°09′59′′N, 0°28′56′′W). Given that Tabarca is a Marine Protected Area, the authors requested and obtained, previously to the sampling, all the necessary permissions from the Fisheries Secretary of the Spanish Ministry of Agriculture, Food and Environment. Fragments (about 5 cm of diameter) of corals, located at 3 m depth, were removed by SCUBA diving using a hammer and chisel, placed into plastic bags underwater, and transported to the laboratory in a cooler. Within 1 to 2 h of collection, each coral fragment was transferred and acclimated for 3 days in a 20-liter aerated aquaria containing filtered (0.45-mm pore size) seawater, before being placed in separate aquaria (1 L) for inoculation experiments. Afterward, fragments were slowly acclimated to the experimental temperature by increasing the temperature by 0.5–1°C per day, until 24°C. The aquaria were illuminated with a fluorescent lamp in cycles alternating 12 h of light with 12 h of darkness. The experiment consisted of 12 aquaria with 2 colonies of coral in each. 3 aquaria with 10^2^ CFU mL^-1^ of *V. mediterranei* VibC-Oc-097, 3 aquaria with 10^5^ CFU mL^-1^ of the AHL-degrading bacterium PQQ-42, 3 aquaria with 10^2^ CFU mL^-1^ of *V. mediterranei* VibC-Oc-097 and 10^5^ CFU mL^-1^ of PQQ-42, and 3 control aquaria without inoculum. The strain PQQ-42 was inoculated 24 h before the *V. mediterranei* VibC-Oc-097.

For plate counts of *V. mediterranei* VibC-Oc-097, 10-fold serial dilutions of sea water from the aquaria (daily sampling) and crushed coral tissue (5 and 10 days after the inoculation of bacteria) were prepared in SFSW and plated on TCBS agar and incubated for 24 h at 25°C.

For tissue extraction, fractions of the colonies were gently washed three times with 50 mL of SFSW to remove non-associated bacteria, broken into small pieces, placed in 50-mL centrifuge tubes, and centrifuged for 3 min at 2,900 × *g*. After centrifugation, the supernatant (coral mucus) was removed and the coral pieces were crushed in SFSW using a mortar and pestle, the CaCO_3_ skeleton was allowed to settle for 15 min, and the supernatant (i.e., crushed tissue) was removed and kept for further analyses (Rubio-Portillo et al., 2014).

The spatial extent of bleaching (white coloration) was estimated after 10 days of inoculation by image analysis using Image-J software (Rasband, 2012) as a percentage of the colony surface area. For chlorophyll a measurements (5 and 10 days after the inoculation of bacteria), 10 mL of the crushed tissue homogenate were centrifuged at 5,000 × *g* for 10 min at 4°C and the supernatant discarded leaving the algal pellet. Pigment extraction was performed with 10 mL of 90% (v/v) acetone, at 4°C during 24 h in the dark, followed by centrifugation at 13,000 × *g* for 10 min and absorbance reading at 750, 664, 647, and 630 nm (Jeffrey and Humphrey, 1975).

### 16S rRNA Gene-based Bacterial Identification

Genomic DNA was extracted as described elsewhere (Martín-Platero et al., 2007). A 1,400 bp fragment of the 16S rRNA gene was amplified using specific primers for *Bacteria* (Lane, 1991): F27 as forward primer (5′-AGAGTTTGATCATGGCTCAG-3′) and 1488R as reverse primer (5′- CGGTTACCTTGTTAGGACTTCA-3′). Amplified PCR products were purified with the Illustra^®^GFX DNA and Gel Band Purification kit (GE Healthcare^®^) and then sequenced directly. The resulting sequences were analyzed with the DNAstar Lasergene Seqman program (Madison, WI, USA). The sequences were identified using the GenBank and EMBL databases (Altschul et al., 1990) and the EzTaxon-e program^1^ (Kim et al., 2012). The 12 sequences determined in this study are available at the EMBL database under accession numbers KT730052-KT730063.

## Results

### Selection of the AHL-Degrading Bacteria

The 450 strains studied in this paper were isolated from a fish hatchery in Salobreña (Granada, Spain) and chosen on the basis of their different colony morphology. We undertook an initial screening of AHL-degradation activity of the isolates in 96-well microtiter plates in the presence of commercial AHLs with fatty-acid chains of different lengths (C_6_-HSL and C_10_-HSL; see Materials and Methods). The AHLs remaining in the media were detected using the biosensor strains *C. violaceum* CV026 (**Supplementary Figure S1**), which produces violacein in the presence of AHLs with short and medium fatty-acid chains; and *C. violaceum* VIR07 (**Supplementary Figure S1**), which originates a violacein pigment in the presence of C_10_-HSL. We found that supernatants associated with 112 strains did not activate the two biosensor strains used which mean that they degraded both C_6_-HSL and C_10_-HSL. These experiments were carried out in triplicate and we obtained the same results. All the experiments were conducted at pH 7 to prevent the lactonolysis of the AHLs (which occurs at basic pH) and the inactivation of biosensors, giving false positive results (Yates et al., 2002).

As a second screening, a well diffusion agar-plate assay of the above 112 selected bacteria was then conducted in the presence of seven commercial AHLs (C_4_-HSL, C_6_-HSL, C_8_-HSL, C_10_-HSL, C_12_-HSL, 3-oxo-C_12_-HSL, and 3-OH-C_10_-HSL). The result was the selection of 12 strains with the ability to degrade a wide range of AHLs. As shown in **Table 1** the 12 strains degrade medium- and long-chain AHLs more or less efficiently, including unsubstituted, oxo- and hydroxyl-substituted AHLs. Nevertheless, none of them show QQ activity against C_4_-HSL.

**Table 1:** Quorum quenching (QQ) activity of the selected marine bacteria and taxonomical identification.

Strain	C_4_-HSL	C_6_-HSL	C_8_-HSL	C_10_-HSL	C_12_-HSL	3-oxo-C_12_-HSL	3-OH-C_10_-HSL	Taxonomic identification	% Identity
PQQ-1	-	++	+	++	++	++	-	*Pseudoalteromonas paragorgicola*	99.78%
PQQ-5	-	++	+	++	+	++	-	*Pseudoalteromonas tetraodonis*	99.7%
PQQ-8	-	++	+	++	++	++	-	*Alteromonas stellipolaris*	99.59%
PQQ-18	-	++	+	++	++	++	-	*Pseudoalteromonas carrageenovora*	99.7%
PQQ-20	-	++	++	++	++	++	-	*Pseudoalteromonas atlántica*	99.44%
PQQ-31	-	++	+	++	+	++	-	*Pseudoalteromonas tetraodonis*	100%
PQQ-33	-	++	++	++	++	++	-	*Alteromonas genovensis*	99.6%
PQQ-37	-	++	++	++	++	++	++	*Alteromonas stellipolaris*	99.7%
PQQ-39	-	++	+	++	++	++	-	*Pseudoalteromonas tetraodonis*	99.48%
PQQ-42	-	++	++	++	++	++	++	*Alteromonas stellipolaris*	99.7%
PQQ-44	-	++	++	++	++	++	++	*Alteromonas stellipolaris*	99.7%
PQQ-84	-	++	+	++	++	++	-	*Pseudoalteromonas distincta*	99.7%

### Characterization of AHL Degradation Activity by HPLC-MS

**FIGURE 1:**
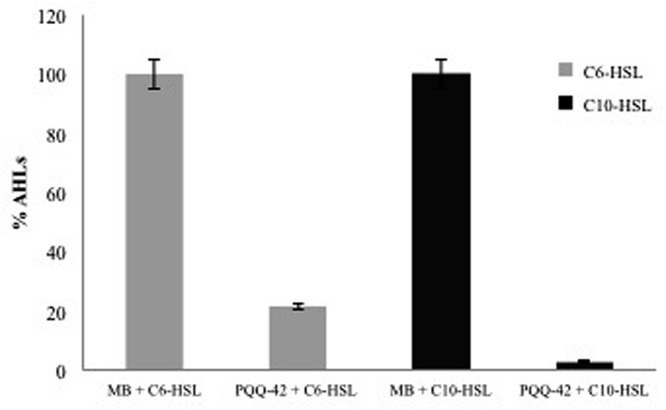
**High-performance liquid chromatography plus mass-spectrometry analysis (HPLC-MS) measurements of C_6_-HSL and C_10_-HSL in the cell-free culture media of the *N*-acylhomoserine lactones (AHL)-degrading strain PQQ-42 after 24 h.** Cell-free MB medium was used as negative control. Initial AHL concentration was 10 μM. Error bars represent one standard deviation.

We applied HPLC-MS analysis to further confirm the degradation of AHLs by the selected AHL-degrading strains. For that purpose we chose a medium- and a long-chain AHL (C_6_-HSL and C_10_-HSL). The results indicated that the 12 selected strains were able to reduce significantly the concentration of both types of AHLs although this capacity was higher against C_10_-HSL (data not shown). Based on these results together with those obtained from the well diffusion agar-plate assays (**Table 1**), the strain PQQ-42 was chosen for subsequent studies. It degraded all AHLs assayed, showing an activity close to 100% in all cases (**Figure 1**).

### Taxonomic Identification of Selected Strains

To identify the 12 AHL-degrading bacteria we determined their 16S rRNA gene sequences. The sequences share high homologies to those of some species of the genera *Alteromonas* (five strains) and *Pseudoalteromonas* (seven strains) (**Table 1**) as compared to reference 16S rRNA gene sequences obtained from the GenBank and EMBL databases using the BLAST search and the EzTaxon-e EzBioCloud program.

### Strain PQQ-42 Degrades AHL Extracts from Pathogenic *Vibrio* spp.

**FIGURE 2:**
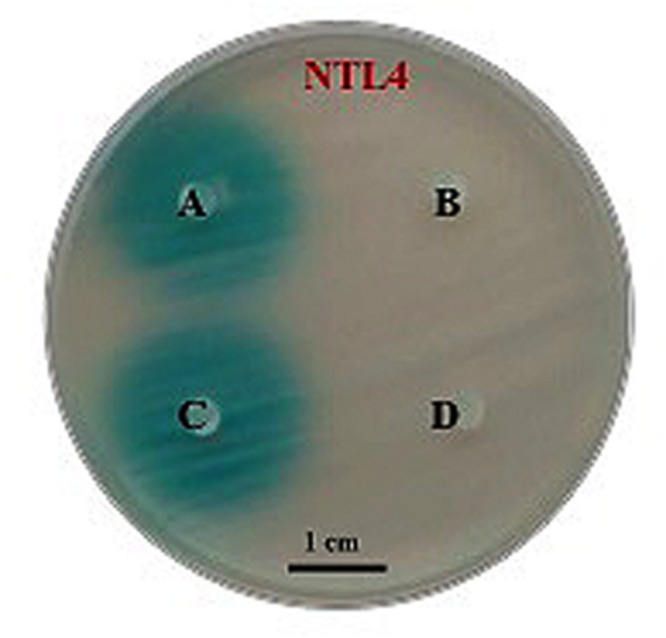
**Degrading activity by strain PQQ-42 of AHLs produced by pathogenic *Vibrio* spp. visualized on an agar plate assay by means of the biosensor strain *Agrobacterium tumefaciens* NTL4 (pZLR4).** Cell-free MB medium added with *Vibrio mediterranei* VibC-Oc-097 AHL extracts (control; **A)**. Culture of strain PQQ-42 added with *V. mediterranei* VibC-Oc-097 AHL extracts **(B)**. Cell-free MB medium added with *V. metschnikovii* NCTC 8483^T^ AHL extracts (control; **C)**. Culture of strain PQQ-42 added with *V. metschnikovii* NCTC 8483^T^ AHL extracts **(D)**. Scale bar, 1 cm.

To ascertain whether strain PQQ-42 could be used *in vivo* to disrupt the QS systems of other bacteria we first assayed its capacity to degrade AHLs produced by aquaculture-associated pathogenic *Vibrio* strains *in vitro*. To this end we assessed the QQ activity of strain PQQ-42 against crude extracts of the four pathogenic strains *V. anguillarum* ATCC 19264^T^, *V. nigripulchritudo* CIP 103195^T^, *V. metschnikovii* NCTC 8483^T^, and *V. mediterranei* VibC-Oc-097. The remaining quantities of AHLs were tested by diffusion agar-plate assays (**Figure 2**) and then analyzed by TLC which confirmed the QQ activity (data not shown). As biosensor strains we used *A. tumefaciens* NTL4 (pZLR4) to detect medium- and long-chain AHLs and *C. violaceum* CV026, which is activated in the presence of short- and medium-chain AHLs (data not shown). We were unable to detect AHL molecules in all tested *Vibrio* crude extracts.

### Type and Location of the AHL-Degradation Activity in PQQ-42

To detect if QQ activity was due to a lactonase-type enzyme, we acidified the supernatants of the cultures of strain PQQ-42 after incubation with the different AHLs tested (C_6_-HSL, C_8_-HSL, C_10_-HSL, C_12_-HSL, 3-oxo-C_12_-HSL, and 3-OH-C_10_-HSL). This acidification allows the lactone ring to restructure itself in the case that it had previously been opened by a lactonase. The recovery of AHL concentration after acidification of the cultures was insignificant in comparison with the negative control (MB added with the same quantity of AHLs and acidified in the same conditions; **Supplementary Figure S2**). This indicates that the degradation activity of the strain PQQ-42 may be due to the activity of an enzyme different of a lactonase.

To determine the cellular location of the enzyme, supernatant and CCE were obtained from the cultures of strain PQQ-42 and AHL-degradation assays were conducted with them in the presence of C_6_-HSL, C_8_-HSL, C_10_-HSL, C_12_-HSL, 3-oxo-C_12_-HSL, and 3-OH-C_10_-HSL. Protein concentration in both supernatant and CCE was previously adjusted to 35 μg mL^-1^. QQ activity of the selected strain was found in the CCE in all of cases but no activity was found in the cell-free culture media (**Supplementary Figure S3**).

### Strain PQQ-42 Degrades AHLs and Reduces Protease Activity and Motility of *Vibrio* Species in Co-culture

In order to carry out co-culture experiments we firstly evaluated whether the selected AHL-degrading strain PQQ-42 interfered with the growth of the four pathogenic strains *V. anguillarum* ATCC 19264^T^, *V. nigripulchritudo* CIP 103195^T^, *V. metschnikovii* NCTC 8483^T^, and *V. mediterranei* VibC-Oc-097 by an antagonism experiment (see Materials and Methods). Our results indicate that PQQ-42 did not have any negative effect on the growth of any of the *Vibrio* strains tested (data not shown). Thus, PQQ-42 was grown in SFSWYE to conduct co-culture experiments with each of the four pathogenic *Vibrio* strains at six different ratios (see Materials and Methods). After 24 h of incubation, the AHLs were extracted from the co-cultures and checked with the biosensor *A. tumefaciens* NTL4. The best results were obtained when 10^2^ CFU mL^-1^ of an overnight culture of any of the *Vibrio* strains assayed were added to an overnight culture of 10^5^ CFU mL^-1^ of PQQ-42. No AHLs were detected in each coculture experiment. This ratio was maintained throughout the co-culture experiments, as was confirmed by plate-counts in TCBS (where *Vibrio* produces yellow-colored colonies) and MA (where *Vibrio* produces smooth white colonies and PQQ-42 produces concave white colonies).

The co-cultures in this ratio were also used to evaluate the effect of the AHL degradation on the virulence factors produced by the four pathogenic *Vibrio* strains. By using different assays (see Materials and Methods), we found that different phenotypes of the *Vibrio* strains were affected under our assay conditions (**Table 2**), such as protease activity and swimming motility of *V. mediterranei* VibC-Oc-097 (**Supplementary Figure S4**).

**Table 2:** Phenotypes of *Vibrio* strains after co-culture with AHL-degrading strain PQQ-42.

Phenotype	*Vibrio mediterranei*	*Vibrio mediterranei* + PQQ-42	*Vibrio anguillarum*	*Vibrio anguillarum* + PQQ-42	*Vibrio nigripulchtritutdo*	*Vibrio nigripulchritudo* + PQQ-42	*Vibrio metschnikovii*	*Vibrio metschnikovii* + PQQ-42
AHLs	++	-	++	-	++	-	++	-
Motility	++	+	++	+	++	+	++	+
Hemolysis	++	+	++	++	++	++	++	++
Protease	++	+	++	++	-	-	++	+
Amylase	-	-	+	++	-	-	+	-
DNAse	+	++	++	++	+	++	+	++
Chitinase	+	-	++	+	+	+	+	-
Lipase (Tween 20)	+	++	++	++	+	++	+	++
Lipase (Tween 80)	++	+	+	++	+	++	++	+
Lipase (Tributyrin)	-	-	-	-	-	-	-	-
Siderophores	-	-	-	-	-	-	-	-
Biofilm	++	++	++	++	++	++	++	++

### Strain PQQ-42 Reduces Virulence of *Vibrio mediterranei* on the Coral *Oculina patagonica*

**FIGURE 3:**
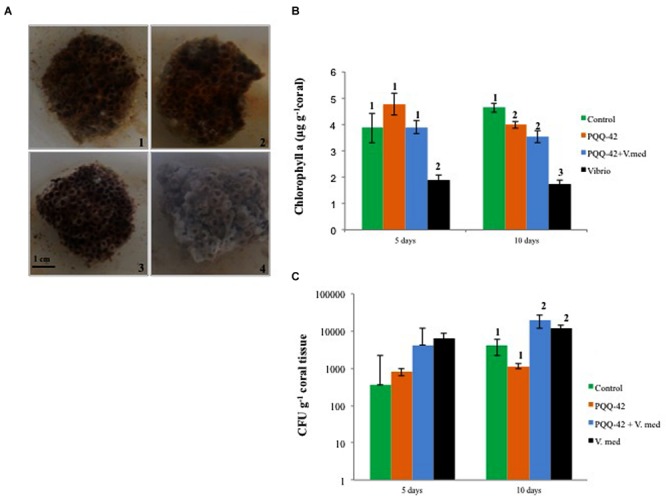
***Oculina patagonica* coral colonies after 10 day’s treatment showing the varying extent of bleaching depending on the bacterium added [1: Cell-free MB medium (control), 2: PQQ-42, 3: PQQ-42+*V. mediterranei* VibC-Oc-097, 4: *V. mediterranei* VibC-Oc-097].** Scale bar, 1 cm **(A)**. Levels of chlorophyll a **(B)** and number of vibrios (CFU g^-1^ **C)** in the tissue of *O. patagonica* after 5 and 10 day’s treatment. Error bars represent one standard deviation. Groups defined by *post hoc* SNK test after ANOVA (*p*-value < 0.05) were done and are showed by numbers.

*In vivo* assays were conducted on colonies of the scleractinian coral *O. patagonica* using PQQ-42 strain. Corals treated with strain PQQ-42 and *V. mediterranei* VibC-Oc-097 showed less tissue damage than those infected with the *Vibrio* strain alone (**Figure 3A**), as we can see reflected in the levels of chlorophyll a detected in the coral tissues (**Figure 3B**). This result indicates that strain PQQ-42 significantly reduces the pathogenicity of *V. mediterranei* VibC-Oc-097 upon the coral *O. patagonica* showing a lower degree of tissue damage (29.25 ± 14.63%) in its presence, compared to when the coral was infected with *V. mediterranei* VibC-Oc-097 alone (77.53 ± 13.22%). Furthermore, chlorophyll a levels were similar in control corals and in corals treated with strain PQQ-42 (**Figure 3B**), which demonstrated that this bacterium does not produce any damage to *O. patagonica* (**Figure 3A**).

Regarding the number of CFU of *Vibrio* in water (**Supplementary Figure S5**) and in coral tissue (**Figure 3C**), we can see that the addition of strain PQQ-42 did not affect the growth of the pathogenic strain.

## Discussion

Aquatic pathogenic and opportunistic bacteria can completely kill mollusks, fish, and coral populations that are reared in aquaculture facilities, resulting in severe economic loss (Paillard et al., 2004; Lafferty et al., 2015). Although antibiotics can be used to control infections, the prevalence of antibiotic-resistant strains makes this increasingly problematic (Akinbowale et al., 2006; Smith, 2008; Cabello et al., 2013). Global efforts are needed to search for novel strategies to control pathogens in aquaculture and to promote a responsible use of antibiotics to make the industry more sustainable as well as to maintain a healthy environment (Defoirdt et al., 2011). In the case of pathogens which depend on QS to regulate virulence, such as some species of *Vibrio*, interference of the AHL signal molecules through QQ presents a good alternative for controlling pathogenicity rather than reducing pathogen number directly by killing them (Defoirdt et al., 2004; Bjarnsholt et al., 2010; Natrah et al., 2011; Zhao et al., 2015). In fact, several studies have demonstrated the potential application of this strategy to control bacterial diseases in aquaculture (Tinh et al., 2008; Nhan et al., 2010; Chu et al., 2014; Romero et al., 2014) and numerous patent applications have been described (Romero et al., 2012). Moreover, it is thought to be more effective and to develop less resistance mechanisms than antimicrobial strategies, since it applies less selective pressure on the pathogens and attenuates bacterial infections without decreasing growth, in contrast to antibiotics (Bjarnsholt et al., 2010; Rasko and Sperandio, 2010). Nevertheless, several authors have recently found the existence of resistance mechanisms against well-characterized compounds with QQ activity (Defoirdt et al., 2010; García-Aljaro et al., 2013, 2016; Kalia et al., 2014) involving enhanced eﬄuxs or modifications on the receptor-binding site of the LuxR-type regulator (Maeda et al., 2012). However, even if resistance mechanisms exist, some authors still think that this fact does not guarantee that it will spread, and moreover, the resistance rates will be much lower than what has been seen for conventional antibiotics (Defoirdt et al., 2011).

Thus, following this beneficial approach, in this study we have selected 12 out of 450 strains from a fish hatchery in Granada (Spain), which were able to degrade a wide range of synthetic AHLs as demonstrated by well-diffusion agar-plate assays. They all grow best at about 2.5–3% (w/v) NaCl, so they are classified as marine bacteria (Kushner and Kamekura, 1988). They belong to *Alteromonas* and *Pseudoalteromonas*, two genera of bacteria commonly found in marine environments and in which QQ has already been described (Romero et al., 2011; Torres et al., 2013). The percentage of isolates with QQ activity found in this study was similar to that found in our previous study (Torres et al., 2013) and is in concordance with those found in previous studies in which it has also been demonstrated that AHL-degrading bacteria are more frequent in marine than terrestrial environments (Dong and Zhang, 2005). Moreover, the strains isolated in this study show QQ activity for medium and long-chain AHLs but they were not effective against short-chain AHLs (C_4_-HSL), a characteristic common amongst marine isolates as revealed in other studies (Romero et al., 2011; Torres et al., 2013).

As a procedure for screening for bacteria with QQ activity, some authors have used enrichment cultures containing AHLs as sole sources of carbon and nitrogen (Leadbetter and Greenberg, 2000; Park et al., 2005; Chan et al., 2009). In our case, two sequential screenings have been performed to select 12 strains with AHL-degrading activity. The first screening for QQ bacteria was carried out using a rapid method modified from the one described by García-Aljaro et al. (2012). It was based on the use of bacterial biosensors using a double-layer microplate assay developed in the presence of medium- or long-chain AHLs (C_6_-HSL or C_10_-HSL). As a second screening, we have done a well diffusion agar-plate assay in the presence of seven different AHLs. These screening procedures have been used in a previous study with successful results (Torres et al., 2013).

Since our efforts were directed toward disrupting QS systems in aquaculture pathogens, the PQQ-42 strain was chosen for further studies since it showed the strongest QQ activity amongst the 12 strains selected in all the assays conducted. It degraded efficiently medium- and long-chain AHLs, including unsubstituted, oxo- and hydroxyl-substituted AHLs. Moreover, we also tested its capacity to interfere with AHL-containing crude extracts of important and highly virulent aquaculture-related pathogens such as *V. anguillarum* ATCC 19264^T^, *V. nigripulchritudo* CIP 103195^T^, *V. metschnikovii* NCTC 8483^T^, and *V. mediterranei* VibC-Oc-097, considered obstacles for many aquaculture settings due to the frequency of outbreaks, geographic distribution, and number of species affected (Toranzo and Barja, 1990; Milton et al., 1997, 2001; Kushmaro et al., 2001; Thompson et al., 2001; Rosenberg and Falkovitz, 2004; Goarant et al., 2006; Sakai et al., 2007; Laganà et al., 2011; Actis et al., 2011). In all cases AHL degradation was complete, indicating that the PQQ-42 strain is able to inactivate AHLs synthesized by pathogenic bacteria. This result was also confirmed when we conducted co-culture experiments *in vitro* in which the strain PQQ-42 was grown in higher concentration with pathogenic *Vibrio* strains (ratio 10^5^:10^2^ CFU mL^-1^). The same results were obtained when the assays were carried out in both marine broth and SFSWYE medium. It has been reported that QQ activity is higher when bacteria are grown in marine water, which shows that the enzymatic activity is improved by the oligoelements that are present in it (Romero et al., 2011).

To determine whether the QQ enzyme of PQQ-42 was an acylase or a lactonase, we carried out acidification and incubation of the samples in the presence of the AHLs. There was no significant recovery of the AHL molecules assayed, suggesting that the degrading activity observed is not due to lactonolysis and may well be due to the activity of some other enzyme, such as an acylase or oxidoreductase. In fact, as far as this is concerned, the presence of acylases in *Alteromonas* has been described in other studies (Romero et al., 2011; Torres et al., 2013). In an effort to identify the AHL-degrading enzyme of PQQ-42, its genome is now being sequenced in collaboration with Dr. Yves Dessaux (Institute for Integrative Biology of the Cell, Gif-sur-Yvette, France) and Dr. Teik Min Chong (University of Malaya, Kuala Lumpur, Malaysia). This approach will provide us with information about a possible gene involved in the QQ activity and then the expression and purification of its protein will allow us to characterize its enzymatic function. In respect to the localization of the degrading activity of the selected strain, supernatant, and CCEs were obtained from cultures of the PQQ-42 strain and they were tested against different AHLs. In all of the cases the results indicated that the QQ activity in this AHL-degrading strain is cell bound, as are most of the acylases and lactonases that have been described so far (Uroz et al., 2005; Romero et al., 2014). Thus, no QQ activity was found in the supernatants of the PQQ-42 cultures.

In this study we have proved that the AHL degradation originated by the strain PQQ-42 in the co-culture experiments has an effect upon the virulence factors produced by *Vibrio* spp., such as the decrease of the production of chitinase and protease and the reduction of swimming motility in all the strains tested. Similarly to our results, the attenuation of some virulence factors (such as protease, biofilm and hemolysis) after co-culture with an AHL-degrading bacterium has also been reported by Chan et al. (2009). Based on these results, *in vivo* experiments have been carried out and demonstrate that the addition of the strain PQQ-42 prevented the bleaching caused by *V. mediterranei* VibC-Oc-097 in the coral *O. patagonica* without reducing vibrio numbers. These findings are of great interest in aquaculture, since among the many marine animals susceptible to vibriosis, corals represent an especially sensitive case. *Vibrio* infections, together with increase of seawater temperatures due to global warming, can led to bleaching and tissue loss in corals (Hoegh-Guldberg, 1999). Other authors have also reported that AHL-degrading bacteria increase the survival rate of turbot larvae (*Scophthalmus maximus*; Tinh et al., 2008) and of giant freshwater prawns (*Macrobrachium rosenbergii*; Nhan et al., 2010). Recently, it has been shown that the oral administration of *Bacillus* sp. QSI-I significantly protects zebrafish from *Aeromonas hydrophila* infection (Chu et al., 2014). The using of organisms or extracts with AHL-degradation capacities as disease control strategy in aquaculture has become of great interest during recent years. One advantage of this strategy may well be the ability to incorporate biocontrol bacteria in the rearing water or by bioencapsulation in the feed stock (Grandclément et al., 2016). Another advantage of the enzymatic degradation of AHLs is that a wide range degradation activity will allow the interference with a higher number of signaling systems, whilst antagonists are usually species-specific.

This study indicates that the addition of the strain PQQ-42 may significantly reduce cumulative mortality of corals and probably of other aquaculture-related species challenged by the pathogenic *Vibrio* species. These properties make the PQQ-42 strain, identified as *Alteromonas stellipolaris*, a feasible and economical way of decreasing *Vibrio* infection and a promising QQ tool for aquaculture.

## Author Contributions

MT, EQ, and IL has carried out the isolation, screening, selection, and identification of the *N*-acylhomoserinelactone-degrading strains. They have also made the characterization and analysis of the type of enzyme as well as the cocultures with pathogenic *Vibrio* strains. They have been in charge of the writing of the paper. ER-P, JA, and AR-E have conducted the coral infection experiments.

## Conflict of Interest Statement

The authors declare that the research was conducted in the absence of any commercial or financial relationships that could be construed as a potential conflict of interest.

## Footnotes

1 http://www.ezbiocloud.net/eztaxon

## Supplementary Material

FIGURE S1 **Assay in a 96-well microtiter plate to detect *N*-acylhomoserine lactones (AHL) degradation using biosensor strains.** Degradation of synthetic AHLs (10 μM) after 24 h’s incubation was revealed by the suppression of violacein production in *Chromobacterium violaceum* CV026 **(A)** and *C. violaceum* VIR07 **(B)**.

FIGURE S2 **Detection of remaining AHLs in the culture media using the biosensors *Agrobacterium tumefaciens* NTL4 (pZLR4), *C. violaceum* CV026, and *C. violaceum* VIR07.** Upper side of each plate: Cell-free MB medium (control; **A)** and strain PQQ-42 **(B)**; bottom side of each plate: Cell-free MB medium (control; **A*)** and strain PQQ-42 **(B*)** after acidification to pH 2. Initial AHL concentration was 10 μM.

FIGURE S3 **Localization of the quorum quenching (QQ) enzyme in the strain PQQ-42 using the biosensors *Agrobacterium tumefaciens* NTL4 (pZLR4), *C. violaceum* CV026, and *C. violaceum* VIR07.** Cell-free MB medium (negative control; **A)**; crude cell extracts **(B)**; and supernatant **(C)** of strain PQQ-42. Initial AHL concentration was 10 μM.

FIGURE S4 **Degradation of AHLs **(A)**, suppression of protease activity **(B)**, and reduction of swimming motility **(C)** of the pathogenic strain *Vibrio mediterranei* VibC-Oc-097 (1) when co-cultured for 24 h with strain PQQ-42 (2)**.

FIGURE S5 **Numbers of *V. mediterranei* VibC-Oc-097 in water**.
