# Supplementary figures and images for: Selection of the N-Acylhomoserine Lactone-Degrading Bacterium Alteromonas stellipolaris PQQ-42 and of Its Potential for Biocontrol in Aquaculture
Source: Front Microbiol. 2016 May 9;7:646. doi: 10.3389/fmicb.2016.00646 (PMC4860449; doi:10.3389/fmicb.2016.00646)

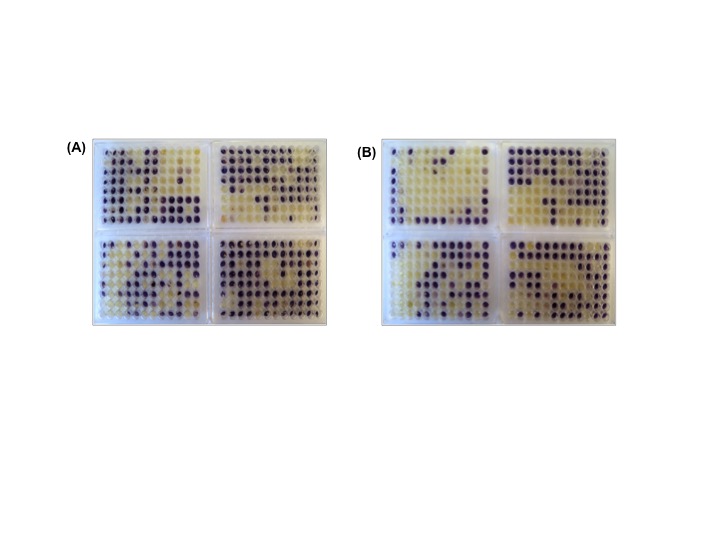

Supplement: FIGURE S1 — Assay in a 96-well microtiter plate to detect N-acylhomoserine lactones (AHL) degradation using biosensor strains. Degradation of synthetic AHLs (10 μM) after 24 h’s incubation was revealed by the suppression of violacein production in Chromobacterium violaceum CV026 (A) and C. violaceum VIR07 (B). [file Image_1.JPEG]

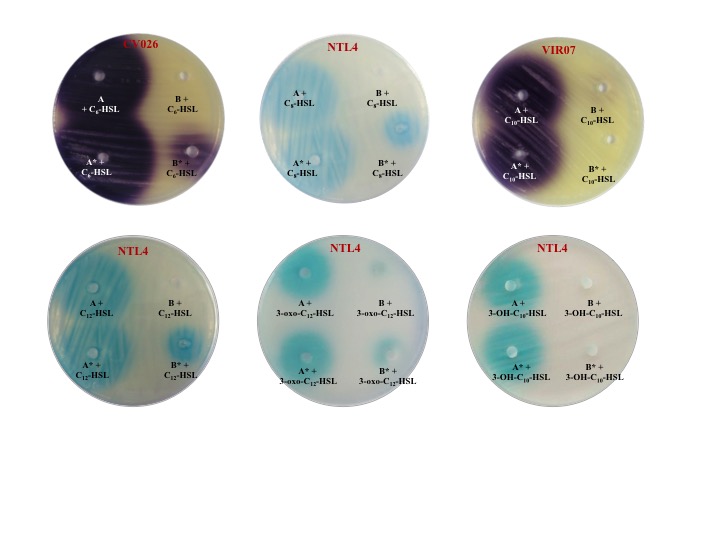

Supplement: FIGURE S2 — Detection of remaining AHLs in the culture media using the biosensors Agrobacterium tumefaciens NTL4 (pZLR4), C. violaceum CV026, and C. violaceum VIR07. Upper side of each plate: Cell-free MB medium (control; A) and strain PQQ-42 (B); bottom side of each plate: Cell-free MB medium (control; A*) and strain PQQ-42 (B*) after acidification to pH 2. Initial AHL concentration was 10 μM. [file Image_2.JPEG]

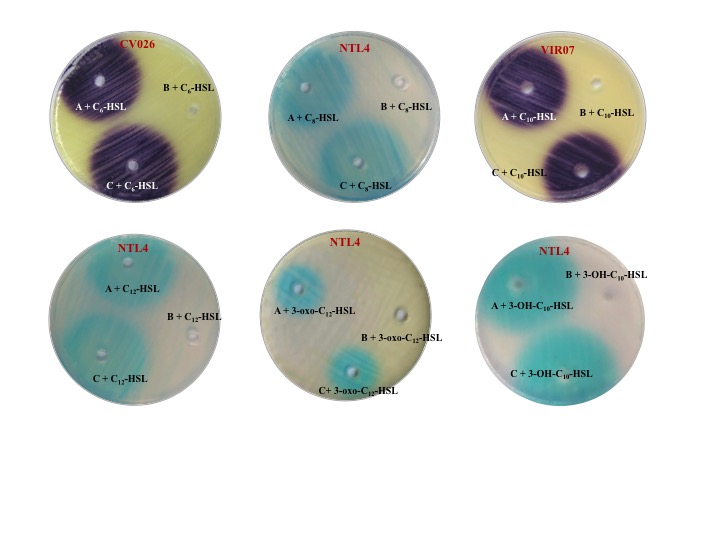

Supplement: FIGURE S3 — Localization of the quorum quenching (QQ) enzyme in the strain PQQ-42 using the biosensors Agrobacterium tumefaciens NTL4 (pZLR4), C. violaceum CV026, and C. violaceum VIR07. Cell-free MB medium (negative control; A); crude cell extracts (B); and supernatant (C) of strain PQQ-42. Initial AHL concentration was 10 μM. [file Image_3.JPEG]

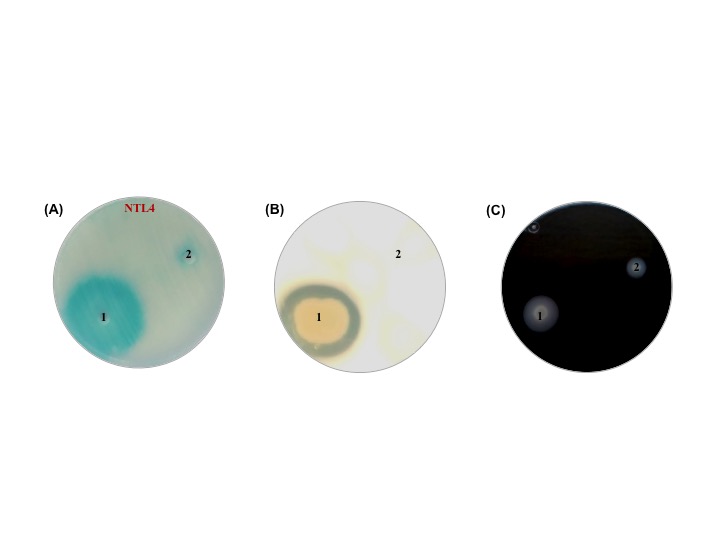

Supplement: FIGURE S4 — Degradation of AHLs (A), suppression of protease activity (B), and reduction of swimming motility (C) of the pathogenic strain Vibrio mediterranei VibC-Oc-097 (1) when co-cultured for 24 h with strain PQQ-42 (2). [file Image_4.JPEG]

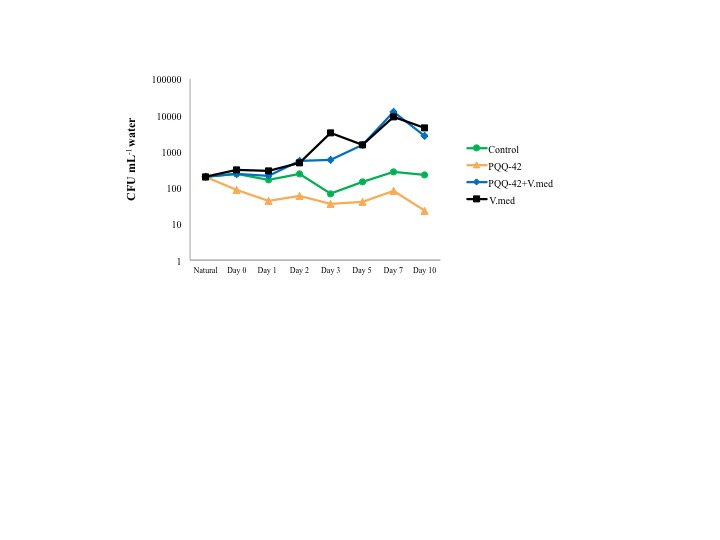

Supplement: FIGURE S5 — Numbers of V. mediterranei VibC-Oc-097 in water. [file Image_5.JPEG]
